# Biotechnological valorization of cashew apple juice for the production of citric acid by a local strain of *Aspergillus niger* LCFS 5

**DOI:** 10.1186/s43141-021-00232-0

**Published:** 2021-09-17

**Authors:** Adekunle Olusegun Adeoye, Agbaje Lateef

**Affiliations:** 1 Department of Food Science, Ladoke Akintola University of Technology, PMB, 4000 Ogbomoso, Nigeria; 2 Laboratory of Industrial Microbiology and Nanobiotechnology, Department of Pure and Applied Biology, Ladoke Akintola University of Technology, PMB, 4000 Ogbomoso, Nigeria

**Keywords:** *Aspergillus niger*, Cashew apple juice, Cheese, Citric acid, Fermentation, Valorization

## Abstract

**Background:**

This work investigates the production of citric acid from cashew apple juice, an abundant waste in the processing of cashew, using a local strain of *Aspergillus niger* and the application of the citric acid as a coagulant for the production of soy cheese. Fungal isolates were obtained from a cashew plantation in Ogbomoso, Nigeria, using potato dextrose agar. Further screening was undertaken to determine the qualitative strength of acid production by the fungi on Czapek-Dox agar supplemented with bromocresol green, with the development of yellow zone taken as an indication of citric acid production. Thereafter, the best producing strain was cultivated in a cashew apple juice medium.

**Results:**

Out of 150 fungal isolates generated from the cashew plantation, 92 (61.3%), 44 (29.3%) and 14 (9.3%) were obtained from cashew fruits, soil and cashew tree surfaces, respectively. Different strains of fungi isolated include *Aspergillus niger*, *A. flavus*, *A. foetidus*, *A. heteromorphus*, *A. nidulans* and *A. viridinutans*. The isolates produced yellow zonation of 0.4–5.5 cm on modified Czapek-Dox agar; the highest was observed for a strain of *A*. *niger* LCFS 5, which was identified using molecular tools. In the formulated cashew apple juice medium, the citric acid yield of LCFS 5 ranged 16.0–92.8 g/l with the peak obtained on the 10th day of fermentation. The citric acid produced was recovered using the double precipitation method with Ca(OH)_2_ and H_2_SO_4_ having ≈ 70% purity of citric acid on HPLC. The citric acid acted as a coagulant to produce soy cheese with 66.67% acceptability by panelists.

**Conclusion:**

This work has extended the frontiers of valorization of cashew waste by a strain of *A. niger* to produce citric acid in high yield, with potential application in food industries.

## Background

The knowledge of microbial biotechnology has aided the utilization of microorganisms for the production of enzymes, vaccines and disease diagnostic tools and the development of new industrial catalysts and products from fermentation that include organic acids [45]. The development of new microbial agents for products of importance to human being was dated to the era of traditional microbial biotechnology [23]. For thousands of years, microorganisms have been used to produce products such as bread, beer and wine. Understanding of control fermentation during the World War I resulted in the development of the acetone-butanol and glycerol fermentation, and from similar processes, the discovery of citric acid production was achieved [13, 23]. Filamentous fungi are extensively used in the fermentation industry for the synthesis of numerous products that include enzymes, functional foods and organic acids [2, 12, 28, 46–48] and more recently in nanobiotechnology to produce nanoparticles [29–31]. One of the most important is the fungus *Aspergillus niger*, used industrially for the production of organic acids [3, 20, 37, 49, 74].

Many microorganisms have been screened for the production of citric acid including bacteria such as *Bacillus licheniformis*, *B. subtilis* and *Corynebacterium* spp. [43] and fungi such as *Aspergillus niger*, *A. awamori* and *A. foetidus* as well as some strains of *Penicillium* such as *P. simplicissinum* and *P. restricium* [3, 10, 40, 60]. Yeasts such as *Candida lipolytica*, *C. intermidia*, *Yarrowia lipolytica*, *Candida tropicalis*, *Pichia kluyveri* and *Saccharomyces cerevisiae* [13, 18, 19, 39] have also been used to produce citric acid.

Among industrially important microorganisms are the Aspergilli; they are a fascinating group of fungi that exhibit immense ecological and metabolic diversity [22]. Out of the group, *A. niger* is regarded as safe with a GRAS status by the US Food and Drugs Administration under the Federal Food, Drug and Cosmetic Act [24, 61, 62]. *A. niger* is used as cell factory for a wide range of commercial enzymes as well as the production of millions of tons of organic acids [16]. It is easy to handle and has the ability to ferment a variety of cheap raw materials to produce citric acid with potentials of high yield [3, 57, 66, 70].

Citric acid has immense applications in different industries that include the production of foods, drinks and pharmaceuticals [73, 75]. It is accepted as an acidulant and preservative that can be consumed in large quantities due to its low toxicity. Citric acid is produced in large amount and it is the next largest fermentation product after ethanol [72]. Its global demand was in excess of 2 million tons in 2015 [20], which requires innovative approaches for production through fermentation to meet the increasing demand. The global value of citric acid was estimated at $3.6b in 2020 [15]. Hu et al. [41] summarized the strategies to enhance the efficient production of citric acid, and these include continuous screening for producers, production of high yielding mutants, exploitation of low-cost substrates, metabolic engineering and optimization of the fermentation process. Thus, the production of citric acid through fermentation will continue to be a hotbed of scientific endeavours. Several cheap agro-industrial wastes that include cassava peel, banana peel, rice straw, orange peel, sugarcane bagasse, chicken feather and pomegranate peel among others have been employed for the microbial production of citric acid [3, 27, 53, 57, 60]. It is envisaged that the list of agrowastes employed in citric acid production will be on the increase as researches in this area progress.

**Fig. 1:**
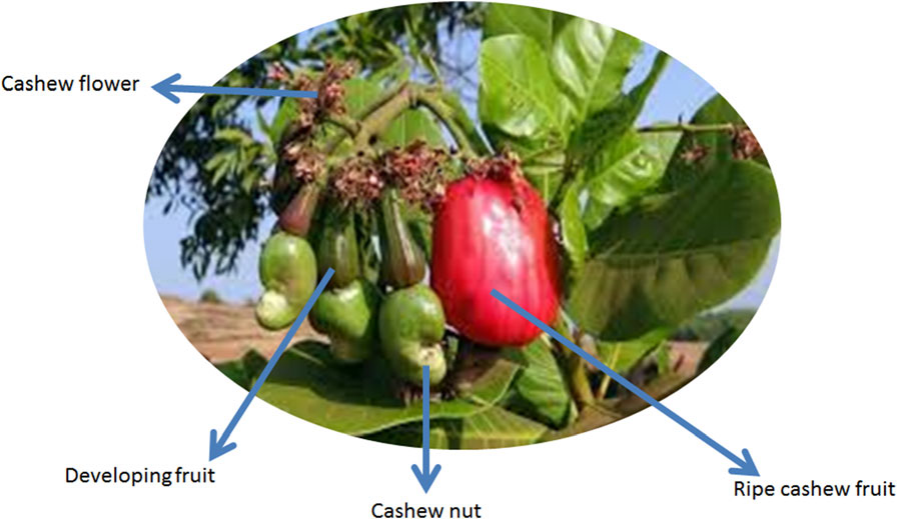
Cashew plant showing the cashew apple fruit

In Nigeria, cultivation of cashew (*Anacardium occidentale*) (Fig. 1) is on large scale with the production of cashew nut estimated at 100,000 tonnes [32] and world production standing at 3.96 million tonnes [69]. The harvesting and processing of cashew nut generate a lot of wastes, which include cashew apple juice, shell, press cake and nut shell liquid [33, 52, 59, 65]. When the fruit matures, the nut falls off the tree with the apple that contains the juice. In actual fact, only about 30% of apple fruit is consumed as food, the remaining 70% goes as waste [42]. The fruit is limited in human consumption by the astringency of the juice due to the high occurrence of tannin that it contains and high perishability [21, 55]. This necessitates the need to valorize the wastes for biotechnological production of valuable products. The cashew apple bagasse and juice have been used to produce bioproducts such as biohydrogen, biosurfactants and bioelectricity [33, 54, 67].

The cashew apple juice constitutes about 90% of the weight of the leftover after the removal of the nut from the whole fruit [54]. The richness of the juice in nutrients [7] makes it an ideal complex medium for microbial growth, which can be explored in the production of citric acid. This innovative approach seeks to add value to cashew production to valorize the apple juice to produce valuable bioproduct (citric acid) that is in high demand worldwide, to reduce the burden of environmental pollution and creation of breeding sites for insects and pathogens occasioned by indiscriminate on-the-farm disposal of apple pomace. Apple juice has been previously explored to produce oligosaccharides, ethanol and oxalic acid through fermentation [14, 56, 68]. In this study, new isolates of *Aspergillus niger* obtained locally from a cashew farm were evaluated for the production of citric acid using cashew apple juice. The biotechnological application of the citric acid produced was investigated in the production of cheese.

## Methods

### Isolation of fungi

The fungal isolates were obtained from cashew apple fruit, the soil and the bark of cashew trees growing on the cashew plantation of LAUTECH Teaching and Research Farm, Ogbomoso, Nigeria. Isolation from the fruit and tree bark was done by wiping moistened sterile swabs, over a surface measured approximately 8 cm^2^ and inoculated on potato dextrose agar (PDA) plates, while the soil sample was serially diluted, and aliquot used as the inoculum. To prevent bacterial growth, 30 mg/l streptomycin was added to the medium, and rosebengal stain was added to prevent faster spore production. The plates were incubated for 7 days at room temperature (30 ± 2 °C ). Later, every reproduced fungal colony was sub-cultured into PDA, Sabouraud dextrose agar (SDA) and Czapek-Dox agar (CZA). These plates were incubated for 7 days at room temperature to obtain the pure cultures of the micro-fungi. The fungi were cultured in an agar slant of PDA and periodically sub-cultured at intervals of two. The cultures were stored at 4 °C until required.

### Screening of fungal isolates for citric acid production

The fungal isolates were screened qualitatively in a Petri dish for the production of citric acid using Czapek-Dox agar that was supplemented with 5 ml of 5% bromocresol green as an indicator. The isolates were inoculated on the plates by inoculating needle using hyphae from a 48-h-old culture and grown at 30 ± 2 °C for 5 days. The production of the yellow zone around the colony was taken as a measure of the ability to produce citric acid, due to the lowering of pH to acidic values [39], and used to rank the fungi by measuring the diameter of the zones. The fungal strains that produced yellow zonation were chosen for further work.

### Characterization and identification of the fungal isolates

The fungal isolates that gave positive results for the production of citric acid in the bromocresol green plate assay were characterized both macroscopically and microscopically. The morphology of the fungi was studied on PDA, while few strands of mycelia were observed under the microscope upon staining with lactophenol cotton blue and identified following standard scheme [26]. The best citric acid-producing strain of *Aspergillus niger* was further characterized using molecular technique employing ITS4 (5′-TCCTCCGCTTATTGATATGC-3′) and ITS5 (5′-GGAAGTAAAAGTCGTAACAAGG-3′) primers [76], following standard protocols [1]. The PCR products were resolved using agarose gel electrophoresis and also sequenced using a commercial facility. The sequences were aligned using BioEdit sequence software, and consensus sequences were deposited in GenBank of the National Centre for Biotechnology Information (http://www.ncbi.nlm.nih.gov/) to identify the isolate.

### Extraction and preparation of juice from cashew apple

The cashew apples were collected and stored in the refrigerator prior to extraction of the juice at 4 °C. They were sorted to remove defective apples. The selected apples were washed to remove dust, dirt and foreign particles. The apples were packed into a weighing can to determine the weight. The fruit juice was then extracted using a juice extracting machine (Cookworks Model no SJ13408 China) and centrifuged at 4000 rpm for 30 min. The clarified juice was then pasteurized at 60 °C for 30 min to denature enzymes that may cause autoxidation and browning reaction during pre-processing storage and to also make it sterile. Furthermore, 0.7 g/l sodium metabisulfite was added to preserve the juice and stored in the refrigerator at 4 °C until use. Earlier, the sugar content of the juice was determined to ensure the maturity of the apple and prevent hypertonic solution that may hinder the growth of the organism by the use of a refractometer [51]. The concentration of sugar was diluted appropriately.

### Production and quantification of citric acid

The fermentation medium was prepared as apple juice that was adjusted to 12 °brix by addition of 10% sucrose and pH adjusted to 6.5 using 0.1 M NaOH. The best fungal strain from the qualitative assay, LCFS 5, was inoculated into the medium following an established procedure [3]. The cultures were incubated at 30 ± 2 °C for 12 days during which the pH, °brix, total titratable acidity and citric acid yield were measured. At each harvest period, the culture was filtered using Whatman filter paper No. 1, and the filtrates used for the analyses. The titrimetric method was used to determine the yield of citric acid as previously described [6] by employing 0.1 M NaOH and phenolphthalein. Briefly, 2–3 drops of phenolphthalein were added to 1 ml of the filtrate which was titrated against with 0.1 M NaOH until the end point is reached with pink colour development. The citric acid yield was calculated thus [25]:

$$ \mathrm{Citric}\ \mathrm{acid}\ \left(\mathrm{g}/\mathrm{l}\right)=0.64\times \mathrm{titre}\ \mathrm{value}; $$

Citric acid equivalent factor is 0.0064 g/l of citric acid

**Fig. 2:**
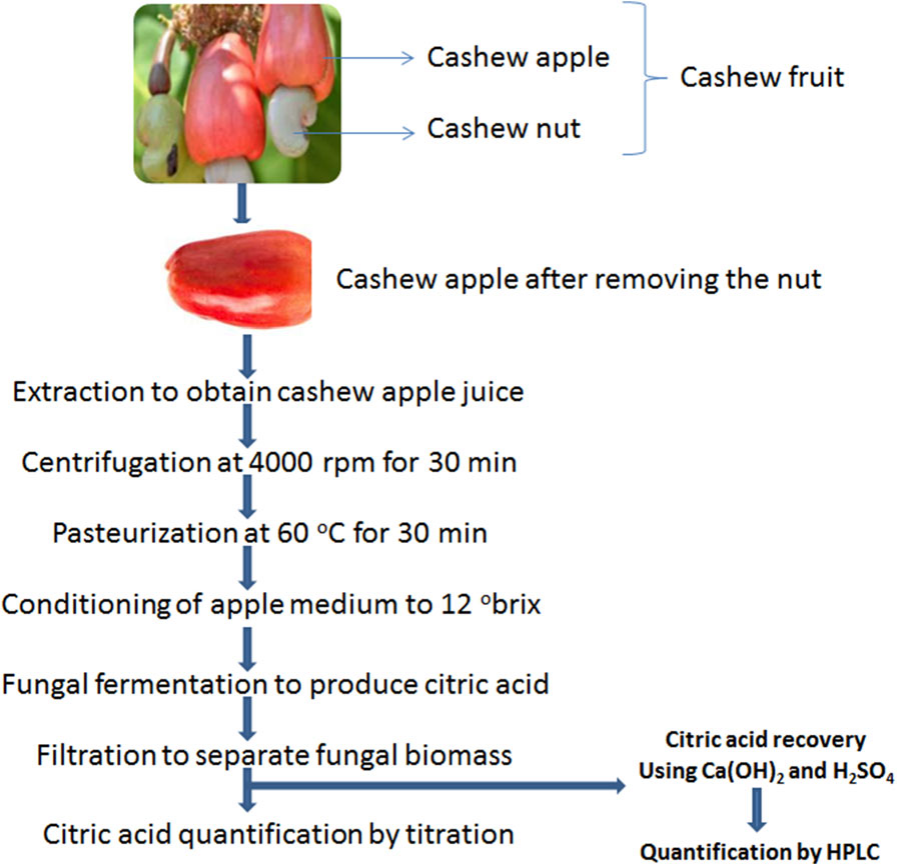
Flowchart of the production of citric acid using cashew apple juice

The flowchart of the production of citric acid from cashew apple juice is depicted in Fig. 2.

### Purification of citric acid

**Fig. 3:**
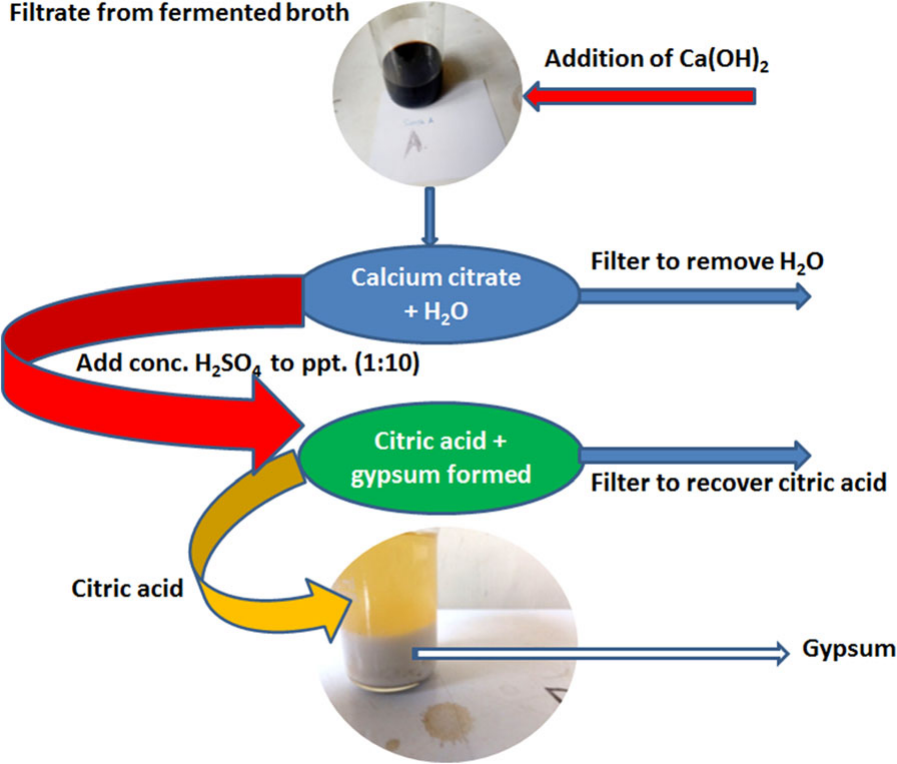
Scheme for the recovery and purification of citric acid

The fermenting broth was filtered using Whatman filter paper No. 1 to remove the fungal biomass. The citric acid in the filtrate was recovered by the double precipitation method [44, 50] using Ca(OH)_2_ and H_2_SO_4_ in a stepwise manner to separate citric acid from the fermenting broth. To 500 ml of the filtrate, 30 g of Ca(OH)_2_ was added and agitated briefly at 60 °C. The calcium citrate precipitate that was formed was recovered, diluted with distilled water (1:10) and further treated with conc. H_2_SO_4_ (10:1). The sequence of the recovery process of citric acid is depicted in Fig. 3. The quantification of the citric acid was done on HPLC Infinity 1260 (Agilent Technologies, USA) [5, 38] at a wavelength of 211 nm using a diode array detector (DAD). The citric acid (Sigma-Aldrich, Darmstadt, Germany) was prepared by dissolving 0.001 g in 10 ml of sodium phosphate buffer and used as standard. Acetonitrile buffer at 70:30 (pH 6.5) was used as the mobile phase at a flow rate of 1.0 ml/min. The temperature of the column (Primesep D, 100 Å, 5 μm, 4.6 × 150 mm) was maintained at 35 °C during the analysis.

### Product development: use of citric acid as a coagulant to produce cheese

Soy cheese was produced using the citric acid obtained from the fungal fermentation of cashew apple juice through the modified methods of Gomaa et al. [36]. Locally procured soya beans were soaked in water (1:5) for 10 min, after which the seed coat was removed manually. The soya bean was milled, mixed with water (1:16) and then sieved using muslin cloth. The filtrate obtained was poured into a clean container and boiled at 100 °C for 30 min, during which citric was added to the boiling liquid until it coagulates, and there is separation of cheese from water. Typically, 16 parts of soy filtrate was treated with 1.2 parts of citric acid. The cheese was collected, pressed in muslin cloth to remove excess water, cut into pieces and steamed with the addition of salt to taste. The control cheese samples were produced using industrial citric acid (0.1 g/ml), and unfermented cashew apple juice under the same condition. Both boiled and fried cheeses produced through this method were presented for sensory evaluation on a 9-point hedonic scale using University undergraduate students as panelists. Nine judges were arranged for sensory evaluation of the cheese produced, to assess qualities which include taste, colour, flavour and texture. The responses of the judges were analysed, and the multiple comparison test was done by employing an analysis of variance.

### Ethics approval and consent to participate

Not applicable

## Results

### Isolation of fungal species

**Fig. 4:**
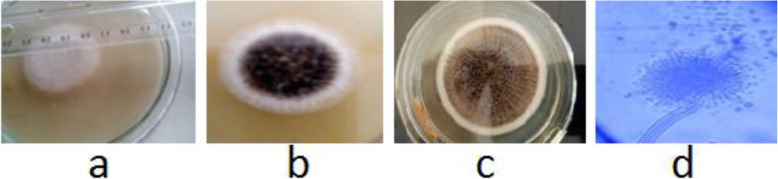
The morphological characteristics of the isolated *Aspergillus niger* (**a** whitish cottony growth at 24 h; **b** black growth at 72 h; **c** loose column at 96 h; **d** microscopic view of the colonial head)

The sampling carried out in LAUTECH cashew plantation produced 150 fungal isolates which were grouped based on sources of isolation. The soil sample produced 44 (29.3%) of the isolates identified, while fruit samples produced 92 (61.3%) of the isolates and 14 (9.3%) were derived from the tree surfaces. Following morphological studies, fungi that were isolated include *Aspergillus niger*, *A. flavus*, *A. foetidus*, *A. heteromorphus*, *A. nidulans* and *A. viridinutans*. Among the isolates, *A. niger* was frequently isolated. The growth of *A. niger* appeared whitish, cotton-like initially at about 24 h of inoculation (Fig. 4a). The mycelia are slender, thread-like, which turned black or blackish-brown with age as shown in Fig. 4b and c. It bears black spores with colonies consisting of a compact white felt covered by a dense layer of dark brown to black conidial heads. Conidial heads were large, globose and dark brown (Fig. 4d), became radiated and tended to split into several loose columns with age as shown in Fig. 4c.

### Qualitative detection of production of citric acid by the fungal isolates

**Fig. 5:**
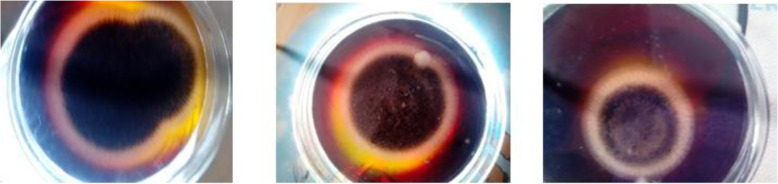
Citric acid production by different strains of *Aspergillus niger* on bromocresol green Czapek-Dox agar showing yellow zonation

**Fig. 6:**
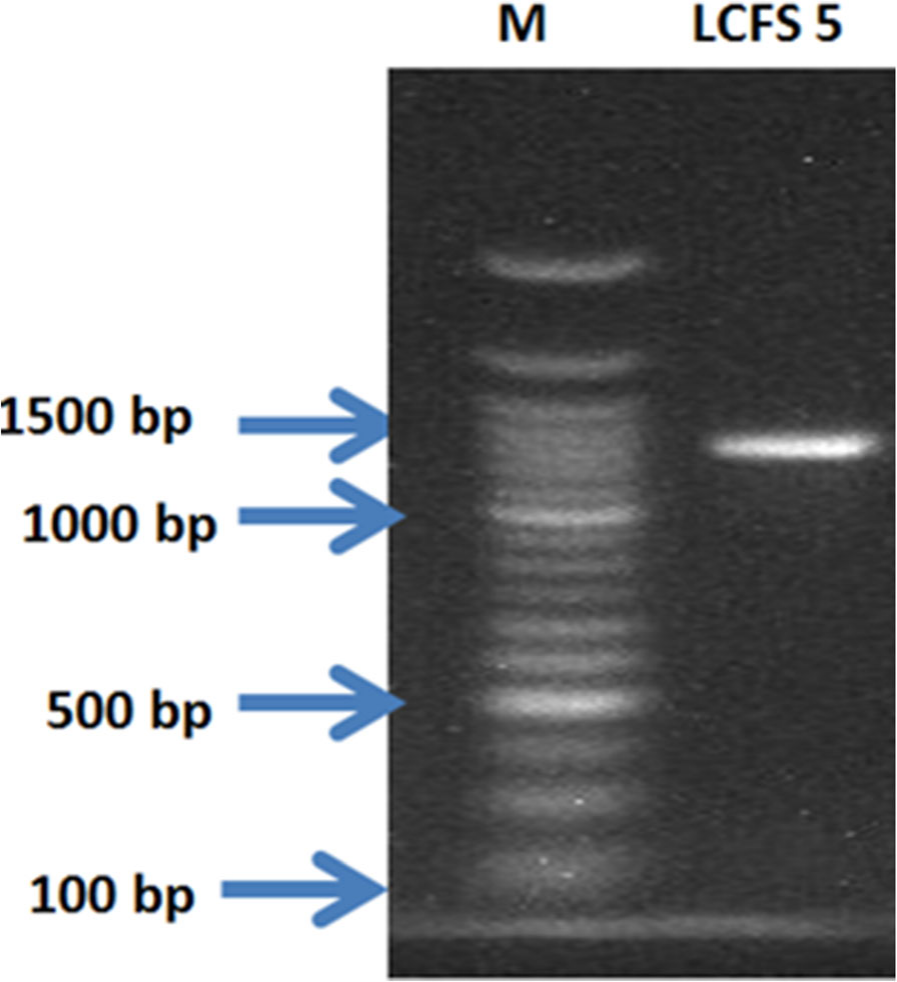
The PCR product obtained via electrophoresis on 1.5% agarose {M, marker; LCFS 5; genomic DNA of *Aspergillus niger* LCFS 5 (MZ448204)}

The fungal isolates produced yellow zonation that ranged from 0.4 to 5.5 cm on CZA modified with bromocresol green, with a strain of *A. niger* LCFS 5 being the best producer of citric acid (Fig. 5). The strain has been identified through molecular technique and its sequence deposited in NCBI with the accession number MZ448204. The gel of PCR products of strain LCF 5 is presented in Fig. 6.

### Production of citric acid in cashew apple juice by *aspergillus Niger* LCFS 5

In a fermentation period of 12 days, *A. niger* LCFS 5 produced 16.0–92.8 g/l of citric acid, with the peak achieved at day 10, and maintained on the 11th day before declining to 90.0 g/l on the 12th day (Table 1). The initial pH of the substrate was 6.5; it declined gradually to reach 2.40 on the 12th day due to the activities of the fermenting organism, while the degree Brix similarly declined during fermentation from the initial value of 12 to 3.8 °brix on the 12th day. Conversely, total titratable acidity increased from 2.50 to reach the peak value of 14.5 on the 10th day of fermentation.

**Table 1:** Quantitative determination of citric acid produced by *A*. *niger* LCFS 5 in the cashew apple juice medium

Day of fermentation	pH	°brix	TTA	Citric acid (g/l)
1	6.50	12.0	2.50	16.00
2	5.40	10.5	3.70	23.70
3	4.43	9.5	6.50	41.60
4	4.33	8.0	7.10	45.44
5	3.73	5.0	12.2	85.00
6	3.70	5.0	12.2	85.00
7	3.50	4.8	13.1	83.84
8	3.48	4.7	13.5	86.40
9	3.47	4.5	14.0	89.60
10	3.44	4.2	14.5	92.80
11	2.51	4.1	14.5	92.80
12	2.40	3.8	14.1	90.00

*LCFS 5* LAUTECH Cashew Farm strain 5, *TTA* total titratable acidity; each value is an average of three readings

### Recovery and purification of citric acid

**Fig. 7:**
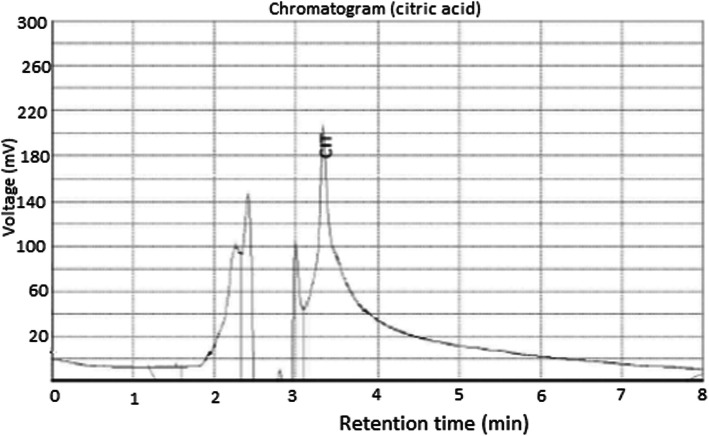
HPLC chromatogram of citric acid produced by *A. niger* LCFS 5 in the cashew apple juice medium

The liquor obtained after fermentation was purified to separate citric acid from impurities as earlier shown in Fig. 3. The HPLC chromatogram of the citric acid is shown in Fig. 7, while Table 2 shows the presence of 69.912 μg/ml of citric acid at a retention time of 3.323 min, representing ≈ 70% composition of the liquor. Unidentified peaks 1–5 had concentrations of 1.3835–12.9518 μg/ml.

**Table 2:**
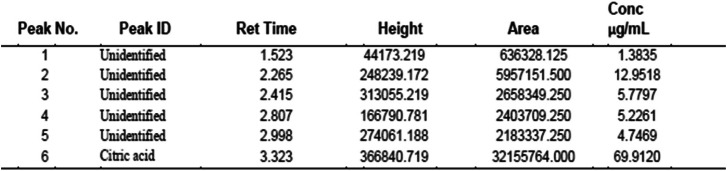
Quantification of citric acid produced using HPLC

### Product development: use of citric acid as a coagulant to produce cheese

**Fig. 8:**
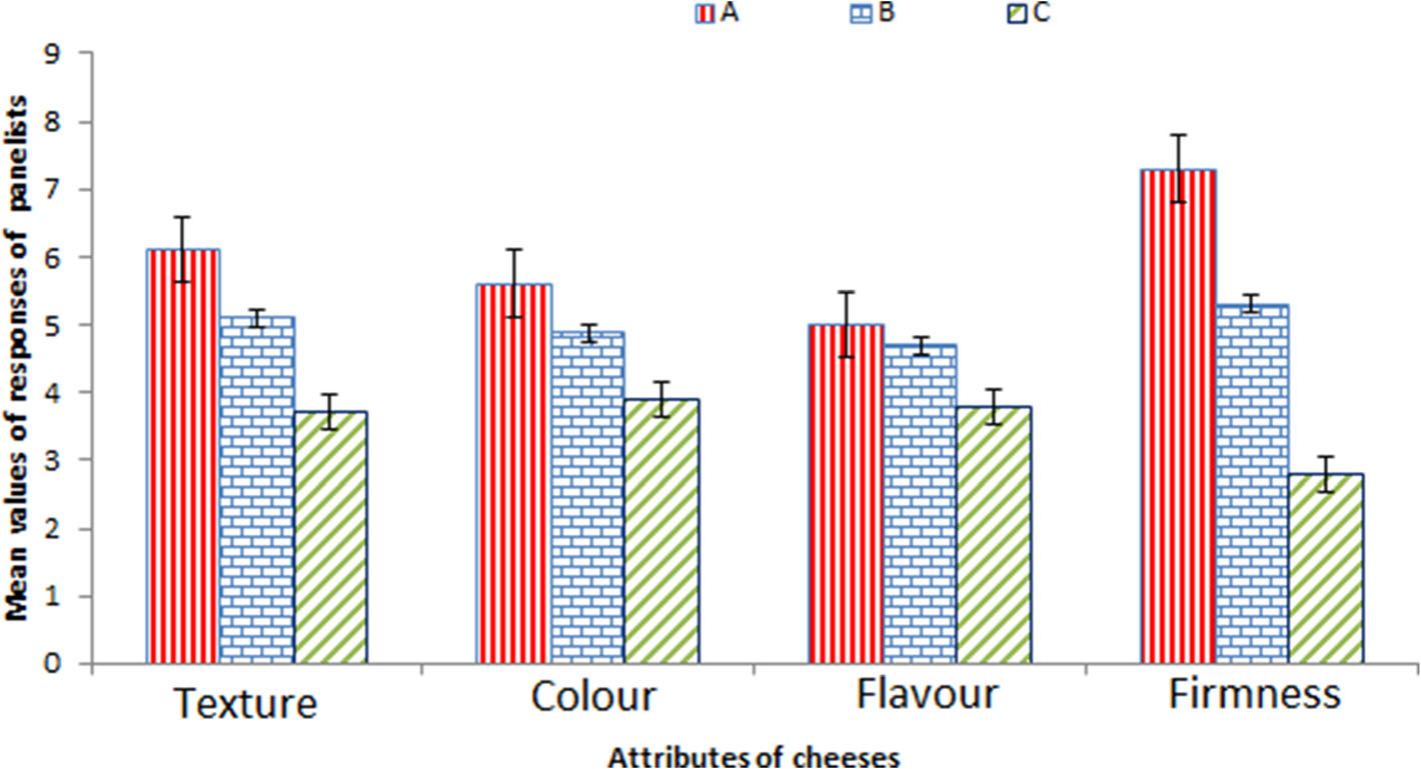
Sensory evaluation of the cheese

The result of quality evaluation of soy cheese is presented in Fig. 8, which showed that sample A produced with the citric acid of *A. niger* LCFS 5 was consistently preferred by the panelists in terms of the texture, colour, flavour and firmness with a performance of 55.56–81.44%. Samples B and C produced using industrial citric acid and unfermented cashew apple juice respectively, followed in terms of the quality.

## Discussion

From the investigation, *A*. *niger* dominated isolation from the fruit possibly due to the presence of sugar and high moisture content. Several authors have reported frequent isolation of *A. niger* from diverse environmental and food samples [11, 35, 63, 64] including those related to cashew [4, 9]. The ability of *A. niger* to produce several hydrolyzing enzymes enables the fungus to utilize a wide range of complex substrates for growth and survival in diverse environments. The cultural morphology of the strains of *A. niger* was in agreement with those previously reported [78, 79] and thus was putatively identified as such.

The qualitative screening of production of citric acid by the fungal isolates was undertaken using the bromocresol green method which has been used by various authors for the rapid screening of fungi for the production of citric. The extent of yellow zonation obtained in this work correlates with zonation of 0.2–9.0 cm previously reported [3, 39, 58] for citric acid producers. The best producing strain *A. niger* LCFS 5 was selected for further work, and its molecular characterization confirmed the fungus as a strain of *A. niger* with accession number MZ448204, having a very high homologous sequence of 99.516% with established strains of *A. niger* in GenBank.

The copious production of citric acid by *A. niger* LCFS 5 to the extent of 92.8 g/l in a cashew apple juice medium is outstanding. For a wild strain of *A. niger*, the yield obtained for LCFS 5 in this study is favourably higher than those previously reported for other strains of *A. niger* grown on different complex substrates such as cassava peel, African star apple peel, palm date, molasses and chicken feather peptone with a productivity of 1.93–68.8 g/l [3, 6, 8, 25, 53, 58]. The good performance of *A. niger* LCFS 5 in producing high amount of citric acid in the cashew apple juice medium may not be unconnected with the richness of the juice in sugars, vitamins, minerals and amino acids [54] that promoted the growth of the fungus. The medium has been exploited for microbial production of ethanol, oligosaccharides, oxalic acid, single cell protein, biosurfactant and biohydrogen among others [14, 54, 56, 68]. Thus, the present study has extended the frontiers of valorization of cashew apple juice to produce citric acid via a biotechnological approach, which adds value to cashew cultivation, and has the potential to address the environmental nuisance that indiscriminate disposal of cashew apple constitutes. This report further lays credence to *A. niger* as a potent producer of citric acid, and strain LCFS 5 adds to the growing list of producer strains that may serve the purpose of extending frontiers in the industrial production of citric acid to meet the high demand for the product.

Citric acid produced by *A. niger* LCFS was successfully recovered through double precipitation and qualitatively detected and quantified using HPLC. While citric acid accounted for almost 70% of the organic acids produced, the five unidentified peaks with about 30% composition of the liquor may be attributed to other organic acids produced by the isolate. In addition to citric acid, *A. niger* is capable of producing itaconic, malic, kojic, succinic and gluconic acids [16, 77] as previously reported. The use of HPLC for quantification of organic acids is a wide practice, as authors have employed HPLC to quantify citric acid production through fungal fermentation with the reported purity of 61.90–65.61% [34, 71]. The result herein presented showed that *A. niger* LCFS 5 has potential for production of citric acid in high titre and comparable to those earlier reported. Therefore, it can be efficaciously deployed for the production of citric acid.

One of the major applications of citric acid in food industry is its use as a coagulant [17]. Hence, the citric acid recovered was used to produce cheese from soy bean. Among the three samples produced using recovered citric acid synthesized by *A. niger* LCFS 5 (sample A), commercial citric acid (sample B) and unfermented cashew apple juice (sample C), sample A was preferred most by the nine panelists in terms of texture, colour, firmness and flavour, where it consistently scored higher than both samples A and B. While sample A had a general acceptance of 66.67%, samples B and C had 55.27 and 39.44%, respectively. The general acceptance of sample A might have been influenced by reduced astringency due to fungal fermentation of tannin in cashew apple juice, the impact of volatile compounds to improve flavour and the firmness that mimics the textural property of meat. Therefore, the citric acid produced by *A. niger* LCFS 5 can find useful application in the production of soy cheese with acceptable attributes.

## Conclusions

This work has established the production of citric acid from cashew apple juice using a high-yielding local strain of *A. niger*, thereby extending the frontiers of biotechnological valorization of wastes from cashew apple processing. The production yield of 92.8 g/l by strain LCFS 5 within 10 days of fermentation is an appreciable yield when compared to those in previous works even under optimized conditions. Further analysis of the fermented cashew apple juice showed purity of the recovered acid at 69.91% showing that cashew apple juice will be a good substrate for the production of citric acid with an acceptable yield. The study has also shown that the utilization of citric acid for the production of cheese can serve as a veritable way of aiding local cheese production as the cheese produced has an acceptable level of 66.67%. Hence, citric acid produced from the fermentation of citric acid can be used as a replacement coagulant for cheese production. This work represents an original contribution to the valorization of agrowastes to produce citric acid in high yield to meet the ever-increasing demand for citric acid worldwide.

## Data Availability

Data used in the experiment are presented in the article.

## Abbreviations

CZA: Czapek-Dox agar
HPLC: High-performance liquid chromatography
LCFS: LAUTECH Cashew Farm Strain
^o^brix: Degree Brix
PDA: Potato dextrose agar
SDA: Sabouraud dextrose agar
TTA: Total titratable acidity
